# Fetal cardiac muscle contractility decreases with gestational age: a color-coded tissue velocity imaging study

**DOI:** 10.1186/1476-7120-10-19

**Published:** 2012-05-09

**Authors:** Nina Elmstedt, Kjerstin Ferm-Widlund, Britta Lind, Lars-Åke Brodin, Magnus Westgren

**Affiliations:** 1 Department of Medical Engineering, School of Technology and Health, Royal Institute of Technology, Stockholm, Sweden; 2 Department of Obstetrics and Gynecology, Centre of Fetal Medicine, Karolinska University Hospital Huddinge, Stockholm, Sweden; 3, Alfred Nobels Allé 10, 141 52, Huddinge, Sweden

**Keywords:** Atrioventricular plane displacement, Longitudinal shortening, Fetal heart contractility, Color-coded tissue velocity imaging, Intrauterine development

## Abstract

**Background:**

Present data regarding how the fetal heart works and develops throughout gestation is limited. However, the possibility to analyze the myocardial velocity profile provides new possibilities to gain further knowledge in this area. Thus, the objective of this study was to evaluate human fetal myocardial characteristics and deformation properties using color-coded tissue velocity imaging (TVI).

**Methods:**

TVI recordings from 55 healthy fetuses, at 18 to 42 weeks of gestation, were acquired at a frame rate of 201–273 frames/s for offline analysis using software enabling retrieval of the myocardial velocity curve and 2D anatomical information. The measurements were taken from an apical four-chamber view, and the acquired data was correlated using regression analysis.

**Results:**

Left ventricular length and width increased uniformly with gestational age. Atrioventricular plane displacement and the E’/A’ ratio also increased with gestational age, while a longitudinal shortening was demonstrated.

**Conclusions:**

Fetal cardiac muscle contractility decreases with gestational age. As numerous fetal- and pregnancy-associated conditions directly influence the pumping function of the fetal heart, we believe that this new insight into the physiology of the human fetal cardiovascular system could contribute to make diagnosis and risk assessment easier and more accurate.

## Introduction

Recent findings demonstrate changes in cardiac muscle contractility in the sheep fetus explained by changes in cardiac troponins with gestational age [1]. Contractility is here correlated with the process of cardiomyocyte binucleation, a transition from mono- to binucleated cardiomyocytes that, as in the human fetus, occur before birth.

Currently there is limited data regarding the functional properties of the human fetal myocardium, and how the human fetal heart works and develops throughout gestation. However, the possibility to analyze the myocardial velocity profile provides new possibilities to gain further knowledge in this area. Color-coded tissue velocity imaging (TVI) facilitates quantitative assessment of myocardial wall motion with a high temporal resolution, which is important considering the high velocity and short duration of the movements of the myocardial walls. In line with other researchers we believe that this technique can add to the evaluation of myocardial function and dysfunction [2] and in gaining new insights into the physiology of the fetal cardiovascular system [3].

The general objective of this study was to evaluate fetal myocardial characteristics and deformation properties using TVI. Particularly evaluating the contractile function and atrioventricular (AV) plane displacement, with the intention to learn if cardiac muscle contractility in the human fetus, as in the sheep fetus, change with gestational age.

## Methods

This study included 55 healthy fetuses at 18 to 42 weeks of gestation, all pregnant women were referred to the Centre of Fetal Medicine at the Karolinska University Hospital Huddinge during 2008–2010. Median gestational age at delivery was 40 weeks (range 34–42), and median birth weight was 3650 g (range 2406–4900), 25 of the fetuses were female and 30 male. All women enrolled were healthy and experienced a normal singleton pregnancy; twin pregnancies and in vitro fertilization (IVF) were excluded. Median maternal age was 30 years (range 18–41). Four of the fetuses were excluded; three due to unsatisfactory fetal echocardiography recordings and one after postnatal data showed maternal pathology.

The study was approved by the Regional Ethics committee in Stockholm, Sweden, and all subjects gave their informed consent to participate.

**Figure 1:**
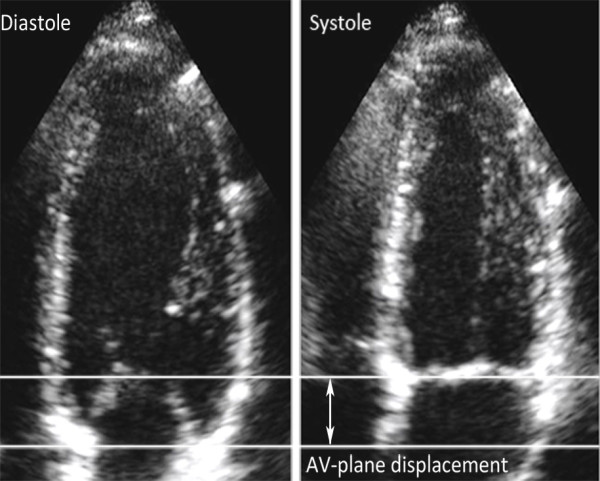
Atrioventricular (AV) plane displacement, the distance covered by the AV plane between the beginning and the end of systole.

Tissue Doppler echocardiography data was obtained with a GE Vivid-i equipment (GE Vingmed, Horten, Norway), using a 3S-RS transducer. All recordings were performed from an apical four-chamber view*.* The angle of insonation to the long axis of the heart was kept as small as possible. The 2D and TVI sector widths were minimized to obtain the highest possible frame rates [4] (201–273 frames/s), and the TVI recordings stored as cine loops of at least 5–10 consecutive cardiac cycles for offline analysis using EchoPAC PC ’08 (GE Vingmed). The peak velocity of early diastolic filling (E’) and atrial contraction (A’) was measured at the basal part of ventricular septum with a sample size of 2 mm. To estimate the AV plane displacement, i.e. the distance covered by the AV plane between the beginning and the end of systole, shown in Figure 1, the time integral of the myocardial velocity curve was measured. The displacement was calculated from an average of 2–3 of the heart cycles stored in the cine loops. The left ventricular (LV) length and width were measured during the end of systole, from base to apex and from left ventricular wall to ventricular septum, respectively.

## Results

**Figure 2:**
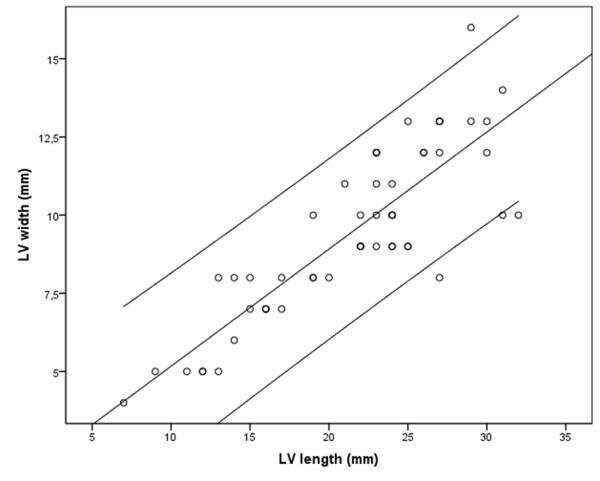
Left ventricular (LV) length and width increased uniformly with gestational age (r = 0.851, p < 0.001). The figure displays a linear regression analysis with 95% confidence interval.

As expected, heart rate (HR) decreased with gestational age, from 144–152 bpm at 18 weeks of gestation to 128–141 bpm at term (r = 0.502). Figure 2 shows that the left ventricle developed uniformly with gestational age (r = 0.851). The LV length increased from 7–12 mm at 18 weeks of gestation to 27–32 mm at term (r = 0.946), and the LV width increased from 4–5 mm at 18 weeks of gestation to 10–16 mm at term (r = 0.851).

**Figure 3:**
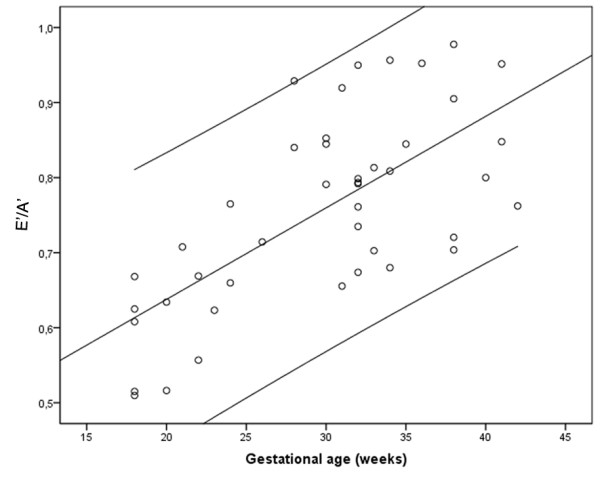
The ratio of the peak velocity of early diastolic filling and atrial contraction (E’/A’) increased with gestational age, (r = 0.688, p < 0.001). The figure displays a linear regression analysis with 95% confidence interval.

The diastolic velocities, E’ and A’, increased with gestational age, even though within larger intervals. E’ increased from 1.3-1.7 cm/s at 18 weeks of gestation to 1.3-3.5 cm/s at term (r = 0.528), and A’ increased from 2.2-3.3 cm/s to 1.9-4.4 at term (r = 0.468). Even so, as Figure 3 shows, E’/A’ is not maintained constant but increases with gestational age (r = 0.688).

**Figure 4:**
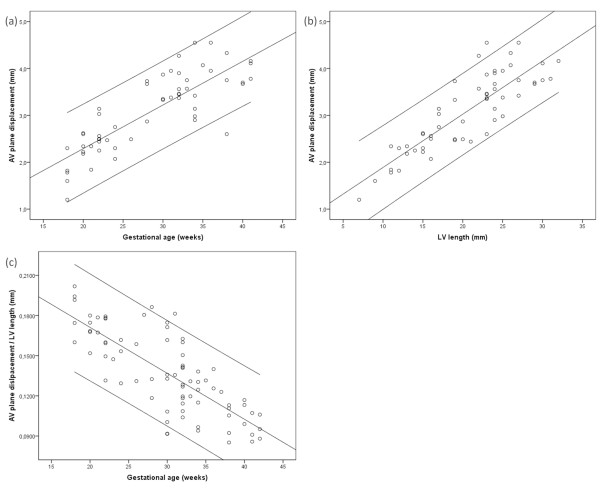
**The atrioventricular (AV) plane displacement increased with gestational age (r = 0.825, p < 0.001) (a), and left ventricular (LV) length (r = 0.851, p < 0.001) (b), while the longitudinal shortening decreased as pregnancy advanced (r = 0.778, p < 0.001) (c).** Figures a-c display linear regression analysis with 95% confidence interval.

As Figure 4 shows, the AV plane displacement increased with gestational age (r = 0.825), in compliance with LV length (r = 0.852). Nonetheless, the ratio of AV plane displacement and LV length decreased as pregnancy advanced (r = 0.778). This longitudinal shortening is seen in Figure 4c. None of the variables measured showed correlation with gender, birth weight or maternal age.

## Discussion

There are limited data regarding the functional properties of the human fetal myocardium. However, the possibility to analyze the myocardial velocity profile provides new possibilities to gain further knowledge in this area. Examining of the myocardial wall motions using TVI allows for direct evaluation of myocardial motion as well as offline analysis using post-processing software. In the present study, using this technique, we show a longitudinal shortening of the fetal myocardium with gestational age, as shown in Figure 4c, which is most likely due to a gestational age-dependent physiological change in the myocardial contractile function.

As the myocardial growth of the left ventricle is uniform, the decreasing HR cannot be due to a change in cardiac movement pattern throughout gestation. Nor can this decrease due to a developed myocardial stiffness, since the E’/A’ ratio increases with gestational age. These findings, together with the conclusion that the displacement of the AV plane is not only dependent on the fetal LV length but also on gestational age, imply a molecular background to the change in cardiac muscle contractility during pregnancy.

Myocardial deformation results from a complex interaction between intrinsic contractile forces, extrinsic loading conditions and elastic properties. Animal studies have indicated that the degree of myocardial deformity reflects local contractile function, preload and afterload of the heart [5]. Since preload is rather stable and afterload declines with advancing gestational age, it is unlikely that changes in load will be the major explanation for the relative decline in myocardial deformation. Rather a change in elastic properties and contractility are plausible explanations for this phenomenon. Most likely, as illustrated in the sheep fetus, contractility is correlated with the process of cardiomyocyte binucleation [1], a transition from mono- to binucleated cardiomyocytes that, as in the human fetus, occur before birth.

The present study demonstrates a linear relationship between LV length and gestational age. Stroke volumes of the ventricles have a curvilinear relationship with gestational age [6] and it seems that the relative high stroke volume of the most immature fetus is explained by increased myocardial deformation. These observations are in agreement with other studies demonstrating declining ejection fractions with advancing gestational age [7,8]. Hypothetically, one could argue that if the immature fetus did not have a myocardium with these favourable elastic properties, the size of the left ventricle and the heart would need to be relatively larger than at term to ensure a constant cardiac output per 100 g of fetal tissue. Thus, since the ratio between the heart and thorax is stable over time and the fetal heart area is approximately one third that of the thorax from approximately 18th week gestation to term, we speculate that the increased contraction with declining gestational age is a prerequisite to ensure these relationships.

It is interesting to speculate how the human fetus can mount an increasing intracardiac pressure with a decreasing deformation of the heart with advancing gestational age. Johnson et al. [9] performed intracardiac punctures in human fetuses, and were able to demonstrate an increase in ventricular systolic and end diastolic pressure with advancing gestational age. Animal studies indicate that the cardiac output does not change in relation to body weight throughout gestation, and the demand of increased circulation to various organs and placenta is met by redistribution rather than an increase in cardiac output [10,11]. Nevertheless, changes to cope with the transition towards LV dominance at birth are not answered for.

The fetal heart allows sustained development under low-oxygen conditions. A decrease in deformation and heart movement might also be an adaptive change to cope with a decline in oxygen supply with advancing gestational age [12]. The birth process will be the ultimate challenge for the fetal heart in regard to oxygen supply and myocardial performance. Fetal cardiac muscle contractility needs to be studied by these means in risk pregnancies. There are some intriguing data indicating adaptive cardiovascular changes in the intrauterine growth restricted (IUGR) or preterm fetus that show increased aortic intima media thickness [13,14], with changes in the modified myocardial performance index (Mod-MPI) [15] and increased coronary blood flow (CF) [16]; all of which correspond to changes of the mechanical pumping function of the fetal heart. We are presently evaluating this method in high risk pregnancies. It is tempting to speculate that myocardial maturation is critical in preparing the fetal heart to cope with the intrauterine environment and the increased demand at birth and to successfully adapt to extra uterine life [1,17].

## Conclusions

Cardiac muscle contractility in the human fetus decrease with gestational age. This is, as illustrated in the sheep fetus, most likely due to changes in the troponin system before and during cardiomyocyte binucleation [1].

As numerous fetal- and pregnancy-associated conditions directly influence the pumping function of the fetal heart, we believe that this new insight into the physiology of the fetal cardiovascular system could contribute to make diagnosis and risk assessment easier and more accurate.

## Competing interests

The authors declare that they have no competing interests.

## Authors' contributions

NE performed the offline measurements, statistical analysis and drafted the manuscript. KFW obtained the tissue Doppler echocardiography data and revised the manuscript. BL and LÅB participated in the design of the study, the interpretation of data and revised the manuscript critically. MW drafted the discussion section, participated in the interpretation of data and revised the manuscript critically. All authors read and approved the final manuscript.
